# Precision oncology in Latin America: current situation, challenges and perspectives

**DOI:** 10.3332/ecancer.2019.920

**Published:** 2019-04-03

**Authors:** Ali Calderón-Aparicio, Andrea Orue

**Affiliations:** Tumor Cell Biology Laboratory, Instituto Venezolano de Investigaciones Científicas IVIC, Centro de Microbiología, Caracas 1020A, Venezuela

**Keywords:** precision oncology, biomarkers, cancer, targeted therapy, access to health care, Latin America

## Abstract

**Background:**

Anti-cancer cytotoxic treatments like platinum-derived compounds often show low therapeutic efficacy, high-risk side effects and resistance. Hence, targeted treatments designed to attack only tumour cells avoiding these harmful side effects are highly needed in clinical practice. Due to this, precision oncology has arisen as an approach to specifically target alterations present only in cancer cells, minimising side effects for patients. It involves the use of molecular biomarkers present in each kind of tumour for diagnosis, prognosis and treatment. Since these biomarkers are specific for each cancer type, physicians use them to stratify, diagnose or take the best therapeutic options for each patient depending on the features of the specific tumour.

**Aim:**

This review aims to describe the current situation, limitations, advantages and perspectives about precision oncology in Latin America.

**Main body:**

For many years, many biomarkers have been used in a clinical setting in developed countries. However, in Latin American countries, their broad application has not been affordable partially due to financial and technical limitations associated with precarious health systems and poor access of low-income populations to quality health care. Furthermore, the genetic mixture in Latin American populations could generate differences in treatment responses from one population to another (pharmacoethnicity) and this should be evaluated before establishing precision therapy in particular populations. Some research groups in the region have done a lot of work in this field and these data should be taken as a starting point to establish networks oriented to finding clinically useful cancer biomarkers in Latin American populations.

**Conclusion:**

Latin America must create policies allowing excluded populations to gain access to health systems and next generation anti-cancer drugs, i.e. high-cost targeted therapies to improve survival. Also, cancer clinical research must be oriented to establish cancer biomarkers adapted to specific populations with different ethnicity, allowing the improvement of patient outcomes.

## Background

Cancer is considered to be a collection of diseases, with their own characteristics derived from specific genetic expression and mutations located in the tissue and cells where it originates [1]. Cancer chemotherapy has always used generic cytotoxic drugs that aimed to inhibit rapid cellular proliferation, a characteristic hallmark of malignant cells. These chemotherapies, although effective at controlling malignant proliferation by inhibiting cellular division, have little precision for specific tumours and often produce high-risk side effects, i.e. development of resistance and immune suppression. Currently, anti-tumour therapies are selected and combined based on their efficacy for particular cancers [2], stratifying cancer treatments based on the tumour-specific features and supporting the concept of personalised medicine of cancer [3, 4]. The National Cancer Institute has defined personalised medicine ‘as a form of medicine that uses information about patient’s genes-proteins signature and environment to prevent, diagnose and treat diseases’ [5].

**Figure 1.**
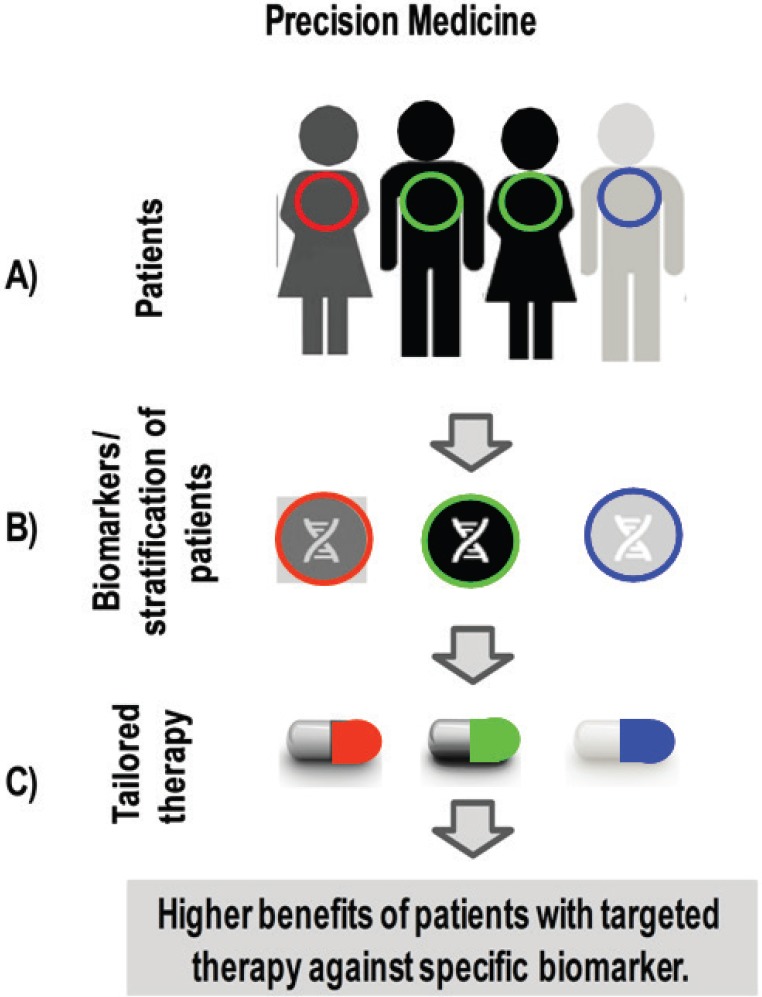
Steps for applying precision medicine in clinic. A) Different patients carry different biomarkers in the same type of cancer. B) Diagnosis of biomarkers allows to stratify patients depending on its specific alteration. C) Targeted-therapies are given to each individual patient to get higher benefits compared to standard therapy.

Often, the terms personalised medicine and precision medicine are used interchangeably. Traditionally, precision medicine referred to specific targeting of molecular abnormalities for diagnosis and stratification of patients who may respond to specific drugs, while personalised medicine has been referred to as the most individualised form of precision therapy, tailored uniquely for each patient [2] (Figure 1). However, there were concerns that the word ‘personalised’ could be misinterpreted to imply that treatments and preventions are being developed uniquely for each person. On the contrary, precision medicine focuses on identifying which approaches will be effective for one group of patients with similar features based on genetic, environmental and lifestyle factors which they share. Therefore, the term ‘precision medicine’ is preferred to ‘personalised medicine’ [6, 7]. In this context, precision oncology refers to the use of patient-specific molecular signatures to perform diagnosis, prognosis, treatment and prevention of cancer [3]. Physicians use these tumour-specific biomarkers to carry out diagnosis, prognosis and delivery of the most recommended treatment decisions to the right patient at the correct dose and time [8].

Many biomarkers predicting the effectiveness of particular treatments have been described for some types of cancer. These biomarkers have been extensively used in clinical practice in developed countries. However, in Latin American countries, the scenario is different, principally due to limited financial and technological resources. In this review, we aim to summarise some of the most promising biomarkers for treating and preventing cancers with high incidence and address the current situation and advances regarding their use in targeted therapies in Latin America.

## Methods

### Literature review

Searches of PubMed with MeSH were carried out between October 2018 and January 2019 using terms specific to *precision oncology, personalised medicine, precision medicine, biomarkers, cancer, molecular targeted therapy, Latin America and access to health care*, and cross-checked with the name of each country of Latin America. Electronic database searches were restricted to English and Spanish. Articles published between 2000 and 2019 were included. To calculate the number of papers studying biomarkers in Latin America, only reports from the region were included (Flowchart 1). Two independent researchers analysed the information and made the conclusions.

## Main body

### Biomarkers and precision oncology

Cancer biology has revealed that each tumour accumulates a unique set of alterations, allowing it to escape from checkpoints that maintain cellular homeostasis [9]. Initially, cancer chemotherapy frequently used cytotoxic drugs that aimed to inhibit proliferative highly cells. However, this clinical approach has little precision and produces high-risk side effects [2]. Therefore, current cancer therapies have moved onto targeting specific features of tumours, improving effectiveness and survival. The high tumour-inter- and intra-heterogeneity bound to the different mutations in each tumour [10] provides a wealth of biomarkers which are used in clinic precision oncology for cancer risk assessment, determination of prognosis and selection of treatment [11, 12] (Tables 1 and 2, biomarkers more widely used in clinic worldwide). This approach has enormously increased during the last years, and diverse consortia have been created for managing all data generated [13], boosting the number of potential targets and associated biomarkers for use in clinical practice [14].

### Types of biomarkers

A biomarker is defined as an objectively measured and evaluated indicator of normal/pathogenic processes or pharmacologic responses to a therapeutic intervention. An ideal cancer biomarker may be useful for different purposes, easily and inexpensively measurable and should identify early-stage disease [15]. Biomarkers are used in precision oncology for the treatment of several types of cancer, i.e. leukaemia, colon cancer, breast cancer, lung cancer and melanoma [16]. These can be divided into groups: (a) **Diagnostic biomarkers:** used to identify and characterise the disease [17], (b) **Predictive biomarkers:** allowing to optimise therapeutic decisions by providing information about the likelihood of response of patients to specific treatments, i.e. alterations in Epidermal growth factor receptor (EGFR), Kristen rat sarcoma viral oncogene homolog (KRAS) and v-raf murine are critical biological determinants of therapeutic response in colon cancer [18], (c) **Prognostic biomarkers:** enable the monitoring of advances of anticancer therapies, the assessment of the stage of tumour and its potential malignancy, as well as the prognosis of disease remission [19] and (d) **Prevention biomarkers:** used to guide individual therapy by identifying patients with different outcome risks (i.e. recurrence of disease) [19]. In Table 1, we summarise some of the most widely used genetic biomarkers for precision oncology in haematological malignancies and solid tumours and its specific alteration.

### Biomarkers in cancer epigenetic

Beside genetic markers, epigenetic markers have also been used for precision oncology. Epigenetic refers to modifications in the genome that do not involve changes in the nucleotide sequence [2]. These changes can be exploited in precision oncology. Different kinds of epigenetic modifications lead to the initial transformation from normal to tumour tissue [20, 21] (Table 2). In general, cancer cells display a global hypomethylation of genome accompanied by focused hypermethylation in dinucleotides CG (cytosine nucleotide followed by a guanine nucleotide), known as CpG islands, in promoters of tumour suppressor genes which become transcriptionally repressed. Conversely, global hypomethylation occurs at the same time in cancer cells and it has been linked with the expression of proto-oncogenes, genomic instability and malignant transformation [22]. Other important epigenetic changes in cancer cells rely on the expression of noncoding RNAs and alterations in patterns of histone methylation and acetylation [21] which alter the transcriptional activity of tumour-development related genes, contributing to cancer progression.

For example, the hypermethylation of Glutathione S-transferase π1 is accurately detected in ~50% of prostate cancer patients [23] and it has been proposed as a diagnostic marker jointly with prostate-specific antigen (PSA) [24]. O6-Methylguanine-DNA methyltransferase*,* an enzyme that promotes the elimination of alkyl-groups from the O6 position of guanine, is the most clinically advanced epigenetic marker in gliomas, where it shows a high grade of hypermethylated CpG sites compared to non-neoplastic tissue, predicting responsiveness to therapy with clinically used alkylating agents as temozolomide and carmustine [25, 26]. The genes Short stature homeobox gene 2 and *SEPTIN9* have also been shown to be hypermethylated in lung and colon cancer, respectively, and their hypermethylation status has been used as a complementary assay for tumour diagnosis in patients through a blood test approved by the Food and Drug Administration [27, 28]. 5-azacytidne and Vorinostab, targeted to inhibit DNA (cytosine-5)-methyltransferase 1 and histone deacetylases, are well-known drugs affecting the chromatin structure and inducing antitumour effects by up-regulating tumour suppressor genes in leukaemia [29, 30]. Thus, the use of epigenetic biomarkers is growing rapidly; even though more studies are necessary to find new targets and gauge their effectiveness in a clinical setting.

### Barriers and economical limitations for applying precision oncology in Latin America

The cancer incidence in Latin America is in general lower than in developed countries, but the mortality is significantly higher. The cancer mortality-to-incidence ratio for Latin America is 0.59, compared with 0.35 in the US [31]. This is in part due to diagnosis at later stages of the disease, as well as barriers for accessing the health system or low quality of health systems in Latin America [32]. Projections to the year 2030 show that cancer cases will increase by 35% in South America and 42% in Mexico [33].

Furthermore, the access of most low-income populations to next generation anti-cancer targeted drugs is limited, mainly due to economic conditions. As well as this, health-care systems in the region are characterised by a lack of coverage for populations excluded from social security or other public financing mechanisms [34] despite that fact that access to healthcare is recognised as a constitutional right in most Latin-American countries [35]. According to The World Health Organization (WHO), 50% of the Latin American population does not have access to high-cost drugs [36]. WHO states that high-cost antineoplastic targeted therapies are essential medicines which should be available in adequate amounts and appropriate dosage forms, at accessible prices [37] and in 2016, three high-cost antineoplastic targeted therapies (imatinib, rituximab and trastuzumab) have been added to the WHO’s Model List of Essential Medicines [33, 37]. Unfortunately, healthcare coverage is not the rule in Latin American countries, and even in those regions where the entitlement to oncology services is guaranteed by law, it is not accompanied by the necessary resources [38]. It means that governments must finance these drugs, allowing people to use them according to the recommended protocols.

Regrettably, anticancer drugs have a higher price in many low- and middle-income countries when compared with higher income nations [39]. A study comparing prices of eight high-cost cancer-targeted therapies found that the United Kingdom paid less for these drugs than Argentina, Brazil, Paraguay and Uruguay. For example, these drugs in the United Kingdom cost 29%–75% less than they cost in Argentina [40]. Another study related to the cost-effectiveness of trastuzumab in Latin America found that, as currently priced, it is not cost-effective in Latin America using the WHO threshold, and to become cost-effective, the price needs to drop between 69.6% and 94.9% [40].

Although some countries in the region have programmes to provide high-cost medicines, e.g. Universal Assurance Program for Explicit Guarantees (AUGE, Chile), Costa Rican Social Security Fund [37], Badan (Drug Bank, Venezuela) and Venezuelan Institute of Social Security (IVSS, Venezuela) nevertheless, these programmes should be continually reviewed, strongly strengthened and continuously updated to guarantee the treatment of the population. In the case of the program IVSS from Venezuela, currently there is a shortage of drugs because of the financial limitations in the country, so the reactivation and development of strategies to improve the distribution of antineoplastic therapies among the population is urgently needed.

On average, the percentage of Latin America’s Gross Domestic Product devoted to health is 7.7% compared with 18% in the United States (ranging from less than 5% in Venezuela and Peru to more than 10% in Costa Rica and Cuba). The overall mean expenditure per new cancer patient in Latin-America is US $7.92 compared with US $183 and US $460 spent by the United Kingdom and the United States, respectively. The overall cost of cancer care has been calculated to represent 0.12% of gross national income *per capita* in South-America versus 0.51% and 1.02% in the United Kingdom and the United States, respectively [36]. These statistics highlight the striking inequity and shortage of resources for cancer care and control in the region [33]. Thus, programmes allowing access of cancer patients to high-cost drugs for precision oncology should be developed to improve survival in Latin America.

### Challenges for implementation of precision oncology in Latin America: much to be done

Characterising the frequency of alterations of cancer-predisposing genes in different types of tumours in Latin America is a first step towards providing useful biomarkers and a precision approach for this disease. The overall cancer profiles regarding Latin American countries are abruptly different because of the genetic mixture between several ethnic groups and different lifestyles [31, 41]. This genetic mixture is reflected in differences in treatment responses between different populations (pharmacoethnicity) [42]. Therefore, from the point of view of public health, diagnostic-therapeutic strategies should be adapted to each population and take into consideration the relationship between ethnicity and types of biomarkers in each population [32]. In some Latin American countries, a wide spectrum of mutations and polymorphisms has been identified in several oncogenes. However, it is necessary to perform a deeper analysis in different Latin American populations to pursue the best biomarkers and therapeutic options for each population. Moreover, oncologists, physicians and all care providers involved in cancer screening, diagnosis and treatment need up-to-date training on how to integrate genomic and molecular data into clinical practice. This represents a great challenge, principally due to large disparities in the level of research among Latin American countries, mainly attributed to funding.

**Figure 2.**
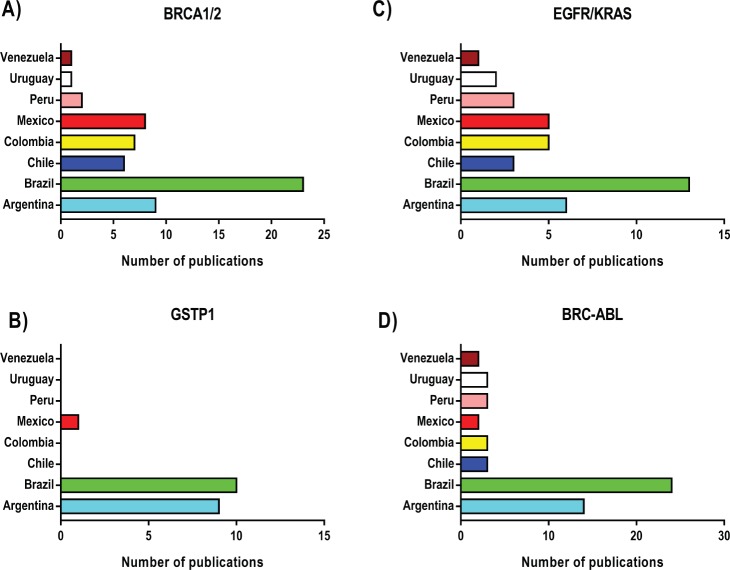
Number of publications development in Latin-America based on use of Biomarkers. These publications correspond to the number of studies published in journals indexed in the PubMed database, between the periods 2000 and 2019. As shown, Brazil does the highest numbers of studies in the field.

In general, Latin America has a low level of investment in research and development compared to developed countries [43]. Excluding Brazil, Chile provides the most public funds for clinical studies in South America, followed by Argentina, with Bolivia, Paraguay and Uruguay providing very little [44]. Figure 2 shows the differences in the number of studies related to some biomarkers used for precision oncology in Latin America. Here, Brazil appears to be the highest contributor for all the markers evaluated. This correlates with the level of expenditure for clinical research between countries in the region since Brazil has the highest budget for research in Latin America. This highlights the importance of the level of funding for clinical research to establish adequate biomarkers for precision oncology in the region.

Furthermore, 65% of all clinical trials in Latin America are sponsored by the pharmaceutical industry (e.g. Mexico, Argentina) [44], which means that Latin American clinical research is highly dependent on funding from these pharmaceutical consortia. Furthermore, public and private hospitals are not adapted to perform clinical research, and the time length for regulatory approval for clinical trial applications is one of the longest in the world, which limits the interest of pharmaceutical companies to conduct clinical trials in Latin America [43].

Another challenge to implementing precision oncology is to overcome the lack of specialised oncologists and diagnoses in rural areas or remote from big cities. The low number of cancer specialists results in an overwhelming workload, as well as a lack of time or interest in clinical research. Besides, there is a lack of educational programmes to motivate health professionals to participate in cancer clinical research, and often these practitioners do not have the necessary training to do this research. In a similar way, the precarious socioeconomic conditions, such as low income or lack of health insurance coverage, limit the access of certain ethnic/racial groups to counselling and genetic testing. For example, the indigenous populations in Latin America have poor health outcomes compared with their non-indigenous counterparts [45].

These issues greatly restrict the development of cancer research in Latin American countries. They highlight the need to develop policies to get patients access to low-cost cancer treatments and to cancer specialists’ care in public centres. These financial constraints are the major challenges for governments and their health systems [33].

Various groups have been working to strengthen the clinical research in the region, such as the Latin American Cooperative Oncology Group, US-Latin-America Cancer Research Network (LACRN), Latin American Federation of Cancer Societies and Latin American Consortium for Lung Cancer Research (CLICaP). CLICaP has developed studies in lung cancer in which more than nine countries in the region participated, detecting genomics differences between populations for mutations in oncogenes:* EGFR, KRAS,* EML4-anaplastic lymphoma kinase*,* Proto-oncogene tyrosine-protein kinase ROS*,* B-cell lymphoma 2 (BCL-2)-like 11 (*BIM*)-deletion and their differences in response to targeted agents [46–48]. For example, an important heterogeneity has been observed in *EGFR* and *KRAS* mutations between Latin American populations [45, 46]. The mutation frequency in *EGFR* was 26.4% but it highly varies according to the population. Notably, the *EGFR* mutation frequency was found to be higher in Peru and lower in Argentina, which could be attributed to differences in ethnicity. Traditionally, Peru has been a destination of Asian migration, where the *EGFR* mutations occur in 30%–50%, contrary to the Argentinean population which has a significant European ancestry, whose mutation rate is 8%–13% [46]. Besides this, the frequency of *KRAS* mutations was lower (14%) than for *EGFR* because both mutations are mutually exclusive [46].

The South American Office for Research and Treatment of Cancer in Southern Brazil has been working with semi-purified plant extracts isolated from South American medicinal plants for potential use as anticancer treatments [36]. As well as this, LACRN, whose aim is to strengthen collaborative research efforts among the participating countries (Argentina, Brazil, Chile, Mexico and Uruguay), is advancing into translational cancer research, including research institutions, hospitals and clinical scientific investigators [49].

Moreover, the Ibero-American network of Pharmacogenetics and Pharmacogenomics seeks to promote precision medicine and research networks in Latin America and the Iberian Peninsula, leading to the inclusion of Latin American populations to analyse their ethnicity, genotype and/or metabolic phenotype in response to therapy (i.e. the MESTIFAR project) [50]. All these efforts should be taken as a starting point to begin establishing precision oncology practices in the clinic.

### Perspectives of precision oncology in Latin America

Currently, the access to up-to-date treatments of cancer in Latin America is complicated by the series of barriers previously mentioned, and the approach to precision oncology is challenging and mainly limited to private practice. In this context, the implementation of not-for-profit programmes for precision oncology is necessary, which should be granted by governments. These programmes should include certain key features: in-depth genomic analysis of the patient’s tumour, interpretation of genomic test results and programmes of access to high-cost drugs for low-income patients to help them to get any molecularly targeted therapy that is prescribed. These programmes should also focus on developing strategies to create networks evolving translational research [51].

As previously mentioned, the incorporation of molecular tests in the coverage of private and public insurance is necessary. A good example is the Oncosalud program, the largest pre-paid system of Peru, which offers free testing with Oncotype DX of breast tumours for their affiliates [52].

On the other hand, the use of liquid biopsies (samples obtained from biological fluids, such as blood, cerebrospinal fluid, semen and others with the aim to detect and evaluate circulating tumour cells, circulating-free DNA, exosomes and other molecules) can aid appropriated patient stratification for targeted therapy and provide important prognostic information. These types of samples have had a great impact on early diagnosis of Non-small cell lung cancer and breast cancer, as well as in other malignant tumours. Unfortunately, its incorporation in clinical practice in Latin America is currently limited [52].

Finally, the education of physicians and healthcare workers must be continually improved to get them prepared for the age of genomic medicine, introducing them to computational methods of genomic analysis in oncology precision [53] in order to obtain highly trained healthcare professionals for precision oncology.

## Conclusion

Latin America is making important efforts in cancer research despite the great deficiencies in funding and disparities in research between countries. Improvements in cancer treatment, including the development of less toxic targeted therapies, improve the quality of life of patients. Unfortunately, these developments are accompanied by increases in cancer treatment costs, making access to these treatments difficult for low-income populations. Additionally, the success of precision therapies requires accurate diagnosis and specialised oncologists. Unfortunately, the lack of specialised doctors in remote or rural areas far from cities restricts access to healthcare for a significant number of people. Therefore, the application of policies that guarantee precise diagnosis, specialised oncology care in public centres and access to treatments at low-costs that allow the administration of cancer therapy in a large proportion of the population are all imperative for the region. Also, Latin America needs an increase of specialised professionals for cancer patient care and updated equipment for cancer treatments. Furthermore, the strengthening and funding of Latin American cancer research centres and networks will also provide good opportunities for the development of cancer research in our countries adapted to the specific features of our populations. Thus, the survival and prognosis of cancer patients in our region could be much improved.

## Conflicts of interest

The authors declare that the research was conducted in the absence of any commercial or financial and nonfinancial relationships that could be construed as a potential conflict of interest.

## Funding statement

This work was financed in part by the Venezuelan Institute for Scientific Research, project Biomarkers and Cancer. The funders did not have a role in the preparation of the manuscript.

## Figures and Tables

**Flowchart 1.**
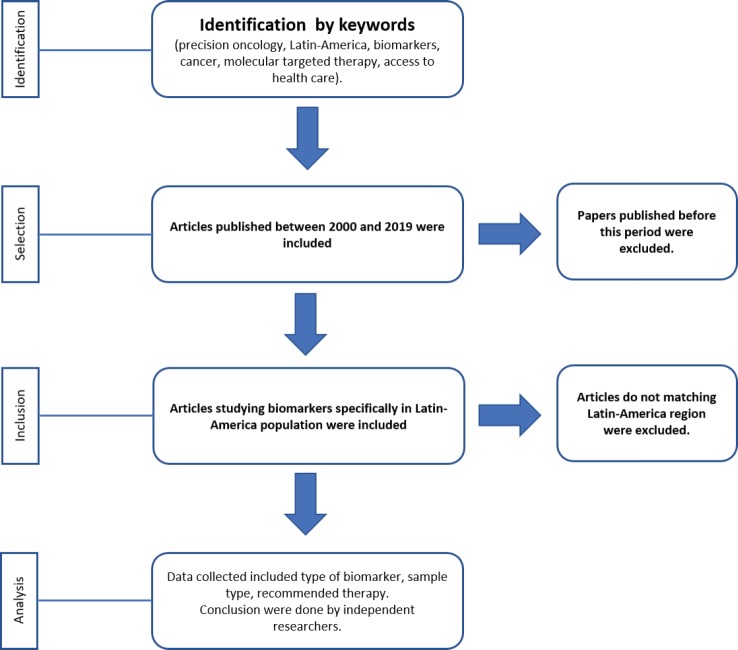
Process for selection of papers working on biomarkers for precision oncology in Latin-America.

**Table 1.** Relevant genetic biomarkers for precision oncology used in clinics worldwide.

Biomarker	Anomalies	Sample type	Therapy	Alteration	Type of Biomarker	Cancer type	Reference
BRCA1/2	Mutation, Deletion	Blood	Poly-ADP ribose polymerase (PARP) inhibitor (Olaparib)	Impaired and DNA repair	Predictive and prognostic	Breast cancer	[54]
HER2/neu ErB-B2	Gene amplification	Tissue	Trastuzumab Lapatinib	Sustains proliferative growth signals	Predictive and prognostic	Breast and gastric Cancers	[55, 56]
ER/PR	Gene expression	Tissue	Tamoxifen	Sustains proliferative growth signals	Primary target of drug. Predictive and prognostic	Breast and ovarian cancer	[57]
EGFR	Gene overexpression	Tissue	Cetuximab Panitumumab	Constitutively activates MEK/ERK pro-growth signalling	Predictive and prognostic biomarker	Colorectal carcinoma (CRC)	[47]
EGFR	Gene mutation	Tissue	Gefitinib Erlotinib Afatinib	Constitutively activates MEK/ERK pro-growth signalling	Predictive and prognostic biomarker	Non-small cell lung cancer (NSCLC)	[58, 59]
HRAS/KRAS (codons 12, 13, 61, 146)	Gene mutation	Tissue	Cetuximab Salirasib	Constitutively activates MEK/ERK pro-growth signalling	Associated with poor response to therapy Prognostic biomarker	CRC, NSCLC, pancreatic cancer	[60, 61]
BRAF	Gene mutation Gene transversion	Tissue	Panitumumab Vemurafenib Dabrafenib	Constitutively activates MEK/ERK pro-growth signalling	Prognostic biomarker	Melanoma, CRC, thyroid cancer	[62]
BCR-ABL	Translocation. Chromosome Abnormality, fusion gene	Blood, bone marrow	Imatinib, Dasatinib Nilotinib, Bosutinib Ponatinib	Compromises fidelity of DNA repair, deregulates proliferation, impairs apoptosis and differentiation	Predictive and prognostic biomarker	Chronic myelogenous leukaemia	[63]
BCL2	Gene expression	Tissue	Venetoclax	Impairs apoptosis	Predictive and prognostic biomarker	Leukaemia, lymphoma, melanoma	[64]
IDH1/2	Gene mutation	Blood	AG120, AG221, AG881	Promote DNA hypermethylation, disrupts differentiation.	Prognostic biomarker.	Acute myeloid leukaemia, gliomas.	[8, 65]
EZH2	Overexpression and overactivation of the gene	Tissue	Tazemetostat	Inhibits apoptosis.	Poor prognostic and predictive biomarker to therapy	Lymphoma, Prostate, NSCLC and breast cancer	[66]
CD20	Loss of gene expression	Blood	Rituximab	Supports B-cell activation and cell cycle progression	Predictive of loss treatment to rituximab	Non-Hodgkin Lymphoma	[67]
ALK	Gene rearrangements	Tissue	Crizotinib, Ceritinib and Alectinib	Creation of a novel fusion protein with transforming activity.	Negative prognostic biomarker and predictive of poor response to TKI	NSCLC	[68–70]
ROS1	Rearrangement	Tissue	Crizotinib	Genes fusions acting as oncogenic drivers	Predictive biomarker to therapy	NSCLC	[71]
Chromosomal bands 11q, 13q, 17p)	Deletion of these Chromosomal bands	Blood	Fludarabine	Non-response to fludarabine	Prognostic and Predictive biomarker to therapy	Chronic lymphatic leukaemia	[72]
AR	Gene mutation	Tissue	Abiraterone, Enzalutamide	Proliferation and progression, activation of PI3K/AKT pathway	Prognostic and Predictive biomarker to therapy	Metastatic castration-resistant prostate cancer	[77, 78]
*AR-V7 (AR3)*	Gene expression	Tissue, Liquid biopsies, circulating DNA	Galeterone	Constitutively activate. Activation of PI3K/AKT pathway, p53 loss, Proliferation and progression	Prognostic and Predictive biomarker to therapy	Metastatic castration-resistant prostate cancer	[78-80]

BRCA1: BRCA1 DNA repair associated; BRCA2: BRCA2 DNA repair associated; ER: oestrogen receptor; PR: Progesterone receptor; EGFR: Epidermal growth factor receptor; HER2-ErB-B2: erb-b2 Receptor tyrosine kinase 2; HRAS/KRAS: Harvey/Kristen rat sarcoma viral oncogene homolog; BRAF: v-raf murine; BCR-ABL: Breakpoint cluster region-Abelson murine leukaemia viral oncogene homolog fusion protein; BCL2: BCL2 apoptosis regulator; IDH1/2: Isocitrate dehydrogenase; EZH2: Histone-lysine N-methyltransferase; CD20: B-lymphocyte antigen CD20; ALK: EML4-anaplastic lymphoma kinase; ROS1: Proto-oncogene tyrosine-protein kinase ROS; AR: Androgen receptor; AR-V7: Androgen receptor, splice variant 7; MEK: mitogen-activated protein kinase kinase 1; ERK: Extracellular Signal-Regulated Kinase; TKI: tyrosine kinase inhibitor; AKT: AKT serine/threonine kinase 1

**Table 2.** Epigenetic biomarkers with potential for precision oncology in clinic worldwide.

Biomarker	Anomalies	Sample type	Therapy	Alteration	Type of Biomarker	Cancer type	Reference
BRCA1/2	Repression transcriptional	Tissue	Poly-ADP ribose polymerase (PARP)-inhibitors	Hypermethylation	Diagnostic biomarker	Breast and ovarian cancer	[26, 73]
GSTP1	Repression transcriptional	Blood and urine	-^1^	Hypermethylation	Diagnostic biomarker of prostate cancer in combination with PSA	Prostate	[24, 25]
MGMT	Repression transcriptional	Tissue	Temozolomide and Carmustine	Hypermethylation	Prognostic biomarker of responsiveness to alkylating agents	Glioma	[26, 27]
EZH2	Mutation and overexpression of EZH2. Repress tumour suppressor genes	Blood	Tazemetostat	Silence tumour suppressor genes by adding H3 K27 methylation	Prognostic biomarker to alkylating agents	Lymphomas	[74]
DNMT	Repression of tumour suppressor genes by DNMT	Blood	Azacytidine	Hypermethylation of tumour suppressor genes	Prognostic biomarker of responsiveness to demethylating agents	Acute myeloid leukaemia	[31, 75]
HDAC	Transcriptional repression	-^1^	Vorinostab, valproate	Delete normal acetylation of histones	Target biomarker of drugs	Leukaemia	[30]
IGFBP3	Transcriptional repression	Tissue	Cisplatin	Hypermethylated promoter	Prognostic biomarker of loss of sensitivity to cisplatin-based therapy	Lung cancer	[76]
SOXH2	Transcriptional repression	Bronchial aspirates	-^1^	Hypermethylation	Diagnostic biomarker for lung cancer	Lung cancer	[28]
*SEPTIN9*	Transcriptional repression	Blood	-^1^	Hypermethylation	Diagnostic biomarker for colon cancer	Colon cancer	[29]

1 Data missing

BRCA1: BRCA1 DNA repair associated; BRCA2: BRCA2 DNA repair associated; GSTP1: Glutathione S-transferase π1; MGMT: O6-Methylguanine-DNA methyltransferase; EZH2: Histone-lysine N-methyltransferase; DNMT: DNA (cytosine-5)-methyltransferase 1; HDAC: Histone deacetylase; IGFBP3: Insulin-like growth factor-binding protein-3; SOXH2: Short stature homeobox gene 2; SEPTIN9: Septin 9
